# Hepatoprotective Effect of Bee Bread in Metabolic Dysfunction-Associated Fatty Liver Disease (MAFLD) Rats: Impact on Oxidative Stress and Inflammation

**DOI:** 10.3390/antiox10122031

**Published:** 2021-12-20

**Authors:** Zaida Zakaria, Zaidatul Akmal Othman, Joseph Bagi Suleiman, Nur Asyilla Che Jalil, Wan Syaheedah Wan Ghazali, Victor Udo Nna, Mahaneem Mohamed

**Affiliations:** 1 Department of Physiology, School of Medical Sciences, Universiti Sains Malaysia, Kubang Kerian 16150, Kelantan, Malaysia; zaida_zakaria@student.usm.my (Z.Z.); zaidaakmal@unisza.edu.my (Z.A.O.); syaheeda@usm.my (W.S.W.G.); 2 Unit of Physiology, Universiti Sultan Zainal Abidin, Kuala Terengganu 20400, Terengganu, Malaysia; 3 Department of Science Laboratory Technology, Akanu Ibiam Federal Polytechnic, Unwana P.M.B. 1007, Ebonyi State, Nigeria; bagisuleiman@student.usm.my; 4 Department of Pathology, School of Medical Sciences, Universiti Sains Malaysia, Kubang Kerian 16150, Kelantan, Malaysia; asyilla@usm.my; 5 Department of Physiology, Faculty of Basic Medical Sciences, College of Medical Sciences, University of Calabar, Calabar P.M.B. 1115, Nigeria; victorudon@unical.edu.ng; 6 Unit of Integrative Medicine, School of Medical Sciences, Universiti Sains Malaysia, Kubang Kerian 16150, Kelantan, Malaysia

**Keywords:** bee bread, oxidative stress, inflammation, NAFLD, MAFLD, NASH, fibrosis, metabolic disorders

## Abstract

Metabolic dysfunction-associated fatty liver disease (MAFLD) is a pathological accumulation of hepatic lipid closely linked with many metabolic disorders, oxidative stress and inflammation. We aimed to evaluate the hepatoprotective effect of bee bread on oxidative stress and inflammatory parameters in MAFLD rats. Twenty-eight male *Sprague-Dawley* rats were assigned into four groups (*n* = 7/group): normal control (NC), high-fat diet (HFD), bee bread (HFD + Bb, HFD + 0.5 g/kg/day bee bread) and orlistat (HFD + Or, HFD + 10 mg/kg/day orlistat) groups. After 12 weeks, the HFD group demonstrated significantly higher body weight gain, serum levels of lipids (TG, TC, LDL), liver enzymes (AST, ALT, ALP) and adiponectin, liver lipids (TG, TC) and insulin resistance (HOMA-IR). Furthermore, the HFD group showed significantly decreased antioxidant enzyme activities (GPx, GST, GR, SOD, CAT) and GSH level, and increased liver oxidative stress (TBARS, NO), translocation of Nrf2 to the nucleus, Keap1 expression and inflammation (TNF-α, NF-κβ, MCP-1) together with histopathological alterations (steatosis, hepatocyte hypertrophy, inflammatory cell infiltration, collagen deposition), which indicated the presence of non-alcoholic steatohepatitis (NASH) and fibrosis. Bee bread significantly attenuated all these changes exerted by HFD feeding. In conclusion, our results suggest that bee bread might have antioxidant, anti-inflammatory, anti-steatotic and anti-fibrotic effects that are beneficial in protecting liver progression towards NASH and fibrosis.

## 1. Introduction

Non-alcoholic fatty liver disease (NAFLD) is identified as a variety of liver disorders ranging from hepatic steatosis (presence of macro-vesicular steatosis only) to non-alcoholic steatohepatitis (NASH) (presence of macro-vesicular steatosis with hepatocyte ballooning, lobular and/or portal inflammation and with/without fibrosis), progressing to advanced fibrosis, cirrhosis, and rarely, may develop into hepatocellular carcinoma [1]. It has been reported that the estimated worldwide prevalence of NAFLD is approximately 25% and this trend is predicted to increase yearly [2]. NAFLD is caused by an excessive intake of dietary fat. Conversely, alcoholic liver disease, which also demonstrates similar hepatic disorders as NAFLD, is caused by an excessive intake of alcohol [3].

The liver plays a vital part in glucose and lipid metabolisms. NAFLD is frequently linked with the main features of metabolic syndromes including obesity [4,5], hyperglycemia [6], insulin resistance [7,8], hyperlipidemia [9] and hypertension [10]. Hence, a new disease named metabolic dysfunction-associated fatty liver disease (MAFLD) has been proposed to replace the old term NAFLD [11,12], and the new term MAFLD is used throughout this study. Enhanced hepatic lipid accumulation, oxidative stress and abnormal inflammatory response remain the foremost pillars in defining the pathogenesis of MAFLD [13,14]. Many studies have linked increased hepatic markers of oxidative stress and a reduction in antioxidant enzymes’ activity with the pathogenesis of MAFLD and NASH [15,16]. Nuclear factor erythroid 2-related factor 2 (Nrf2) is a key transcription factor regulating the antioxidant signaling to defend against oxidative stress in cells [17]. Increased lipid levels in the hepatic tissues result in the increases in lipid peroxidation and Nrf2 expression in the liver [18]. Under the stressful conditions, Nrf2 detaches from Kelch-like ECH-associated protein 1 (Keap1), a Nrf2 repressor protein, and translocates to the nucleus to stimulate the antioxidant response [19]. In the nucleus, Nrf2 forms heterodimers with small musculoaponeurotic fibrosarcoma (Maf) proteins and up-regulates the expression of antioxidant response element (ARE)-containing genes including glutathione peroxidase (GPx), glutathione reductase (GR), catalase (CAT), superoxide dismutase (SOD), glutamate cysteine ligase (GCL), NAD(P)H/quinone oxidoreductase 1 (NQO1) and heme oxygenase-1 (HO-1) [20].

Even though inflammation is crucial for an effective immune response, its dysregulation exerts a fundamental role in the propagation of MAFLD-related metabolic dysregulations [21]. In MAFLD, an impaired inflammatory response is commonly depicted by aberrant levels of cytokines as well as chemokines that are tenacious with enhanced hepatic damage [22]. Under such circumstances, persistent activation of innate immune responses, mostly driven by excessive hepatic lipid accumulation, can stimulate the classic features of pathological liver inflammation [23]. For instance, continuously increased leptin and pro-inflammatory mediators (cytokines and chemokines), for example, tumor necrosis factor alpha (TNF-α), nuclear factor kappa β (NF-κβ) and chemoattractant protein-1 (MCP-1), and reduced adiponectin and anti-inflammatory markers such as interleukin-10 are likely to promote NASH progression and initiate fibrosis formation [23,24,25,26].

To date, there is no specific therapy for MAFLD, although lifestyle interventions such as appropriate food consumption, regular exercise and weight loss have been reported to be effective in managing MAFLD [27]. Still, these treatment options are challenging and difficult to follow due to adherence issues, particularly for long-term management. A variety of pharmacological therapies are available to treat MAFLD such as pioglitazone and vitamin E, nevertheless, these treatments have been reported to have limited efficacy towards MAFLD [28]. Scientists have, therefore, become more captivated in researching antioxidant and/or anti-inflammatory interventions as a useful approach to mitigate this disease progression. The use of bee products as complementary medicines and dietary supplements to heal numerous illnesses has been practiced for many decades. Today, there is a growing interest in exploring their benefits and pharmacological properties for the potential development of nutraceutical and functional food of bee products including honey, bee pollen, royal jelly, propolis and bee bread. Bee bread or ambrosia refers to the fermented pollen mixed with honey and secretions of the bee’s salivary glands and stored in the hive [29,30]. The foraging bees deliver the collected pollen loads to the hive and pack them directly into the empty cells of the honeycomb, where it is later secured and mixed with wax and honey [31]. The lactic fermentation process of pollen in the honeycomb performed by *Lactobacillus* bacteria under anaerobic conditions converts pollen into a more preserved state called “bee bread” [32], which gives it a high nutritive value [33]. Bee bread has been reported to have excellent digestibility and abundant chemical compositions [34]. It contains proteins, vitamins, lipids, microelements, phenolic and flavonoid compounds that are recognized as the added values to its nutritional and therapeutic properties. Bee bread exhibits antibacterial [35], antioxidant [36] and anticancer [37] properties. Previous studies have demonstrated that bee bread possesses hepatoprotective properties as shown by decreases in the severity of liver damage in alcoholic fatty liver disease patients [38], and in aluminum [39] and carbon tetrachloride (CCl_4_)-induced liver injury [40] in rats. Bee bread has also shown its medicinal effects in other varieties of ailments including cardiovascular diseases [41], male fertility [42], renal dysfunction [43] and cancer [44] in in vitro and in vivo studies. Nevertheless, there is only one set of data demonstrating the effects of bee bread on the high-fat diet (HFD)-induced fatty liver disease rat model, which reported that the consumption of bee bread (80, 400 and 800 mg/kg/day) downregulated the expressions of hepatic lipogenic protein and genes such as fatty acid synthase (FAS) and acetyl-CoA carboxylase (ACC) levels, hence inhibiting hepatic lipid synthesis [45]. Therefore, the purpose of this study was to assess the potential alleviative effects of bee bread in protecting against MAFLD or the complications implicated in its aggravation including oxidative stress and inflammation. This study measured the changes in the anthropometrical parameters (body weight gain, BMI and abdomino-thoracic circumferences), serum levels of lipids (TC, TG, LDL and HDL), liver enzymes (ALT, AST and ALP), adiponectin, glucose and insulin resistance, liver lipid contents (TC and TG), liver oxidative stress status, immunoexpressions of redox homeostasis regulators (Nrf2 and Keap1) and inflammation, as well as liver histopathological and fibrosis status.

## 2. Materials and Methods

### 2.1. Bee Bread Preparation

Fresh bee bread (*Heterotrigona itama*) was procured from a local stingless bee farm (Mentari Technobee PLT, Kelantan, Malaysia), dried and grounded to powder before storage at −20 °C until further analysis.

### 2.2. Animals and Diet

The protocol for this study was approved by Universiti Sains Malaysia Institutional Animal Care and Use Committee (USM IACUC) with the following approval number: USM/IACUC/2020/(126)(1109). Twenty-eight male *Sprague-Dawley* rats aged between 8–10 weeks (body weight = 200–230 g) were procured from the laboratory of the Animal Research and Service Centre (ARASC), Universiti Sains Malaysia. The rats were treated humanely as per the guidelines of the National Institute of Health Guide for the Care and Use of Laboratory Animals. Each of the rats was individually housed in a polypropylene cage with sterilized husk bedding in a room on a 12 h light-dark cycle, controlled temperature (22 ± 2 °C) and humidity (55 ± 10%). All the rats were allowed food (standard diet) and water ad libitum during acclimatization period (1 week) prior to the start of the experiments. The rats were either provided with a standard diet (Altromin, Altromin Spezialfutter GmbH & Co. KG, Lage, Germany) or a high-fat diet (HFD) that was formulated based on a previous study [41]. The standard diet consisted of 12% fat, 24% protein and 64% carbohydrate, which provided around 31.8 kcal/g energy. Meanwhile, the HFD consisted of 31% fat, 12% protein and 46% carbohydrate, which supplied around 51.65 kcal/g energy.

### 2.3. Experimental Design

Following acclimatization, all the rats were randomly distributed into four groups with seven animals per group: normal control (NC, received standard diet and distilled water), high-fat diet (HFD, received HFD and distilled water), bee bread (HFD + Bb, received HFD and bee bread at 0.5 g/kg/day) and orlistat (HFD + Or, received HFD and orlistat at 10 mg/kg/day) groups. The dose of bee bread was selected as the best dose based on our previous preliminary study using different doses of bee bread (i.e., 0.5, 1.0 and 1.5 g/kg/day) (*n* = 3/group) for 6 weeks. The results showed that bee bread at 0.5 g/kg/day reduced serum levels of liver enzymes, i.e., aspartate aminotransferase (AST), alanine aminotransferase (ALT) and alkaline phosphatase (ALP), and liver fat deposition compared to the HFD group (unpublished observation). Whereas, the dose of orlistat was chosen according to a previous finding using orlistat at 10 mg/kg/day in HFD-fed rats [46]. Standard diet and HFD were provided ad libitum. Orlistat was obtained from Xepa-Soul Pattinson Sdn. Bhd. (Melaka, Malaysia). Bee bread and orlistat were freshly and separately prepared by suspending in 1 mL of distilled water and then administered to the rats via oral gavage for 12 weeks.

### 2.4. Determination of Anthropometrical Composition

The body weight (g) of each rat was determined every week and the body weight gain was assessed as the difference in the body weight of the rats at the end of the experimental period and at baseline. Meanwhile, the naso-anal length (NAL) or body length, thoracic circumference (TC) perimeter (perimeter of behind the foreleg) and abdominal circumference (AC) (perimeter of anterior to the forefoot) were calculated using a measuring tape. The AC/TC ratio of the rats were calculated while body mass index (BMI) was also determined using the formula: weight (g)/(NAL)^2^(cm^2^), and obesity was defined as BMI value above 0.68 g/cm^2^ [47].

### 2.5. Blood Sampling and Tissue Preparation

Rats were euthanized following 12 weeks of the experimental period (starved overnight, 12 h) by an intraperitoneal injection of a cocktail of ketamine at 90 mg/kg and xylazine at 5 mg/kg. Blood samples were taken from the posterior vena cava into a tube containing gel clot activator and left to clot at room temperature. The blood and serum were separated after being centrifuged at 4000 rpm for 10 min at 4 °C. Serum samples obtained were aliquoted into Eppendorf tubes and kept at −80 °C until use. The adipose (epididymal, peritoneal and perirenal) and liver tissues were removed, washed in ice-cold saline, and blotted to dryness before being weighed (Denver Instrument Company, Arvada, CO, USA) and the liver index was determined. The liver was then divided into two portions: one small portion was used to prepare 10% (*w*/*v*) tissue homogenate. Briefly, the liver samples were weighed and placed in centrifuge bottles added to ten volumes (*w*/*v*) of ice-cold phosphate buffered saline solution (pH 7.4). Then, the samples were centrifuged at 4000 rpm for 10 min at 4 °C, and the obtained supernatant was aliquoted into Eppendorf tubes and stored at −80 °C until further use. Meanwhile, another portion of liver tissue was stored in 10% buffered formalin for immunohistochemical and histopathological assessments.

### 2.6. Evaluations of Serum Lipid and Liver Profiles, and Adiponectin

The serum levels of both total cholesterol (TC) and triglyceride (TG) were assessed using commercially available kits (ARCHITECT c kit, Abbott, IL, USA) via an enzymatic-colorimetric method. Serum low-density lipoprotein (LDL) was determined by referring to a formula reported in a previous study [48], i.e., LDL (mmol/L) = (TC – HDL − (TG/5). Meanwhile, serum high-density lipoprotein (HDL) was evaluated by removal of LDL-Cholesterol, chylomicron, and VLDL-Cholesterol by cholesterol oxidase, cholesterol esterase and catalase, whereas, serum AST, ALT and ALP were assessed using a method by the International Federation of Clinical Chemistry (IFCC), which evaluated the catalytic concentration of reagent enzymes and the contaminants. All liver enzymes were estimated using Abbott Architect Ci8200, Abbott Park, IL, USA. The level of serum adiponectin was estimated using a commercially available kit according to the manufacturer’s instruction (Elabscience, Houston, TX, USA) (Catalog No: E-EL-R3012).

### 2.7. Measurements of Serum Glucose, Insulin and HOMA-IR

The levels of glucose and insulin in the serum were measured using commercially available kits procured from Qayee-Bio Life Science Co., Ltd., Shanghai, China (Catalog No: QY-E11702) and Elabscience Biotechnology Inc. Co., Ltd. Wuhan, Hubei, China (Catalog No: E-EL-R2466), respectively, referring to the manufacturers’ instructions. The homeostatic model of assessment-insulin resistance (HOMA-IR) was evaluated as described by a previous study [49].

### 2.8. Liver Biochemical Analyses

The levels of hepatic TC, TG, tumor necrosis factor alpha (TNF-α), nuclear factor kappa β (NF-κβ), and interleukin 10 (IL-10) were evaluated using commercially available kits obtained from Qayee-Bio Life Science Co., Ltd. (Shanghai, China) (Catalog No: QY-E10860, QY-E11395, QY-E10880, QY-E11356 and QY-E11536, respectively) according to the manufacturers’ instructions. Nitric oxide (NO) level was measured by commercially available kits purchased from Elabscience Biotechnology Inc. Co., Ltd. (Wuhan, China) (Catalog No: E-BC-K035-M). Meanwhile, the concentrations of thiobarbituric acid reactive substance (TBARs), glutathione peroxidase (GPx), glutathione S-transferase (GST), glutathione (GSH), glutathione reductase (GR), superoxide dismutase (SOD) and catalase (CAT) in the liver were measured according to previously described methods [50,51,52,53,54,55,56].

### 2.9. Immunohistochemical Analysis of Nrf2, Keap1 and MCP-1 Protein Expressions

In order to determine the immunohistochemistry (IHC) detections of Nrf2, Keap1 and MCP-1 proteins in the hepatic tissue, the 3 µm paraffin sections were collected and processed as follows: The liver sections were initially deparaffinized and hydrated. The antigen retrieval was achieved by heating the liver sections in tris-EDTA buffer with 0.05% Tween 20 (pH 9.0) solution for 3 min in a pressure cooker, followed by a 5-min cool-down in distilled water. Thereafter, the endogenous peroxidase activity in the liver sections were quenched for 5 min with 3% hydrogen peroxide solution (diluted in phosphate buffered saline) and washed with distilled water and tris-buffered saline containing 0.05% Tween 20 (TBST) (pH 7.4), respectively. Later, the liver sections were incubated overnight at 4 °C with the following rabbit polyclonal primary antibodies: Nrf2 (Cloud-Clone Corp, Katty, TX, USA) (1:100), Keap1 (Cloud-Clone Corp, Katty, TX, USA) (1:100) and MCP-1 (Abcam, Cambridge, UK) (1:100). The following day, the primary antibody was removed by washing with TBST and the liver sections were incubated with Dako EnVision System Labelled Polymer-HRP (Agilent Technologies, Inc., Santa Clara, CA, USA) containing goat anti-rabbit secondary antibody at room temperature for one hour and then incubated with Dako DAB + substrate chromogen (Agilent Technologies, Inc., Santa Clara, CA, USA) (1:1) mixed solution at room temperature for 5 min. The liver sections were then washed under running water to remove any excess DAB and counterstained with hematoxylin (Merck, Darmstadt, Germany). The scoring for Nrf2, Keap1 and MCP-1 protein expressions were calculated by two independent pathologists in a blinded manner as described by a previous study [57]. Briefly, immunoreactive score was evaluated semiquantitatively in which the staining intensity was multiplied with the percentage of positive cells. Staining intensity was scored as follows: 0 (colorless), 1 (light yellow), 2 (brownish yellow) and 3 (brown). Percentage of positive cells was graded as follows: 0 (negative), 1 (10%), 2 (11–50%), 3 (51–75%) and 4 (75–100%).

### 2.10. Liver Histopathological Examination

The liver tissues were fixed in 10% buffered formalin, embedded in paraffin and sliced into 3 µm sections. The liver sections were subsequently stained with hematoxylin and eosin (H&E) (both Merck, Darmstadt, Germany) for assessing the histological steatosis (i.e., macro- or micro-vesicular steatosis and hypertrophy) and inflammation, and then rated according to the NAFLD activity score (NAS) [58,59] as follows: Cases with scores ≥ 5 were diagnosed as NASH and cases with NAS ≤ 3 were reported as non-NASH. Masson’s trichrome staining was used for determining the hepatic collagen deposition and graded for the fibrosis stage [60] as follows: 0 (None), 1 (perisinusoidal or periportal fibrosis), 2 (perisinusoidal and portal/periportal fibrosis), 3 (bridging fibrosis) and 4 (cirrhosis). All histopathological evaluations were carried out by two independent pathologists in a blinded manner.

### 2.11. Statistical Analysis

Statistical analysis was carried out using GraphPad Prism, 8th Version Software (GraphPad Software Inc., San Diego, CA, USA). Normality was assessed by the Shapiro–Wilk normality test, and homogeneity of variance was assessed by the D’Agostino–Pearson Omnibus test. All data are expressed as mean ± standard deviation (SD). One-way analysis of variance (ANOVA) was used for multi-group comparisons and Tukey post-hoc test was used to determine the differences between the groups. Statistical significance is expressed as *p* < 0.05.

## 3. Results

### 3.1. Effects of Bee Bread on Anthropometrical Parameters

The anthropometrical parameters of rats from all four groups are demonstrated in Table 1. Rats in the HFD group demonstrated significant increases in body weight gain, BMI and AC/TC ratio in comparison with the NC group. Conversely, all these parameters were distinctly decreased in the bee bread and orlistat groups except for the AC/TC ratio in the orlistat group compared with those of the HFD group.

### 3.2. Effects of Bee Bread on Serum Lipid and Liver Profiles, and Adiponectin

Table 2 shows the levels of serum lipid, liver profiles and adiponectin in each group. The intake of an HFD significantly increased the serum levels of TG, TC and LDL, and significantly reduced the level of HDL in the HFD group compared with those in the NC group. However, the administration of bee bread and orlistat significantly lowered the levels of TG, TC and LDL. Moreover, the decrease in the level of HDL was successfully attenuated by the treatment with bee bread and orlistat.

Elevation in the liver enzymes is recognized as the first manifestation of liver disease. Hence, to confirm if bee bread could improve liver functional capacity, the serum concentrations of ALT, AST and ALP were assessed in this study. There were significant increases in the serum levels of ALT, AST and ALP after 12 weeks of HFD intake in the HFD group compared to the NC group. In contrast, these levels were notably mitigated by the intake of bee bread and the results were also similar in the orlistat group. In addition, adiponectin plays a part in the pathogenesis of MAFLD and the reduction in adiponectin indicates the progression towards NASH. This study showed a significantly decreased serum level of adiponectin in the HFD group when compared with the NC group, whereas, the level of adiponectin was significantly increased after intake of bee bread. A similar result was also observed in the orlistat group (Table 2).

### 3.3. Effects of Bee Bread on Serum Glucose and Insulin Resistance

Table 3 demonstrates the concentrations of fasting serum glucose, insulin and HOMA-IR in each group. After 12 weeks of HFD consumption, the serum levels of glucose and insulin were markedly increased in the HFD group compared with those in the NC group. Moreover, the HFD group also exhibited a significantly higher HOMA-IR index indicating a substantial increase in insulin resistance. On the contrary, administrations of bee bread and orlistat exerted protective effects as shown by eliminating the increase in serum glucose and insulin concentrations caused by HFD. Consistently, both bee bread and orlistat groups also exhibited a marked decrease in HOMA-IR index compared to the HFD group.

### 3.4. Effects of Bee Bread on Adipose and Liver Tissue Weights, Liver Index and Liver Lipid Contents

As shown in Table 4, the rats in the HFD group demonstrated significant higher epididymal, peritoneal, perirenal and total adipose tissues weights than the NC group, and the intake of bee bread significantly decreased these adipose tissue weights, and similar findings were also demonstrated in the orlistat group. In addition, increases in absolute liver weight and liver index can indicate the buildup of fat in the liver. The results showed that the absolute liver weight and liver index in the HFD group were increased significantly in comparison with the NC group, whereas the administration of bee bread markedly decreased these parameters relative to the HFD group. However, no significant differences were observed for these parameters following treatment with orlistat (Table 4). In addition, the levels of liver TG and TC were found to be significantly increased in the HFD group compared with those in the NC group. In contrast, the intake of bee bread significantly reduced these lipid contents in the liver, and similar findings were also observed in the orlistat group (Table 4).

### 3.5. Effects of Bee Bread on Liver Oxidative Stress Status in the Liver

MAFLD has been connected with oxidative stress and reduced activity of antioxidant enzymes in the liver. To assess the effects of bee bread on liver oxidative stress status, the levels of oxidant and antioxidant markers were determined in the present study. As presented in Table 5, the oxidative stress markers TBARS and NO levels were significantly elevated in the HFD group compared with those in the NC group. Conversely, lower TBARS and NO concentrations were demonstrated in bee bread and orlistat groups than those in the HFD group. Furthermore, the activities of enzymatic antioxidants, namely: GPx, GST, GR, SOD and CAT, and the level of GSH were significantly reduced in the HFD group compared with the NC group. In contrast, administrations of bee bread and orlistat effectively prevented the decreased GPx, GST, GSH, GR, SOD and CAT in these groups after HFD intake.

### 3.6. Effects of Bee Bread on Liver Inflammatory Markers

Inflammation is one of the hallmarks of MAFLD and is used as a marker of advanced MAFLD. The prolonged and continuous intake of HFD can lead to increased inflammation in the liver. Hence, to determine whether bee bread can protect against inflammation, the levels of liver pro- and anti-inflammatory markers (TNF-α, NF-κβ and IL-10) were measured in this study. In Table 6, the results demonstrated significant increases in the liver levels of pro-inflammatory markers TNF-α and NF-κβ and a significant reduction in the level of the anti-inflammatory marker IL-10 in the HFD group compared with the NC group. As expected, the administration of bee bread reversed these changes significantly and were comparable to the orlistat group.

### 3.7. Effects of Bee Bread on Nrf2, Keap1 and MCP-1 Protein Expressions

**Figure 1:**
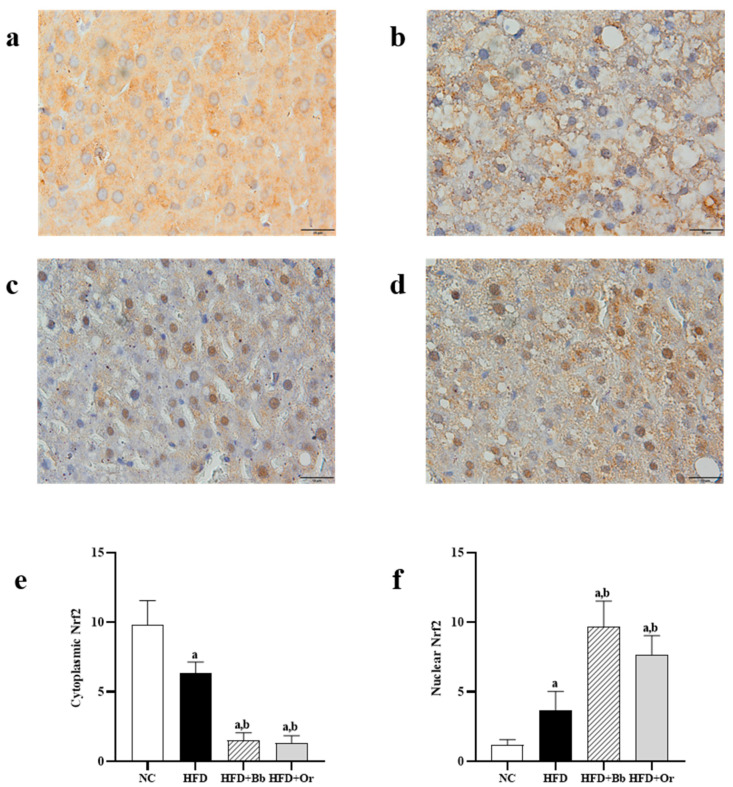
Expression of Nrf2 in liver sections from NC (**a**) HFD (**b**) HFD + Bb (**c**) and HFD + Or (**d**) groups. Magnification, ×1000, scale bars represent 10 µm. The staining intensity of cytoplasmic Nrf2 and nuclear Nrf2 were quantified (**e**,**f**). Values are expressed as mean ± SD, *n* = 7/group. One-way ANOVA, followed by Tukey post-hoc test. ^a^ *p* < 0.05 vs. NC group, ^b^ *p* < 0.05 vs. HFD group. NC, normal control; HFD, high-fat diet; HFD + Bb, high-fat diet + bee bread 0.5 mg/kg/day; HFD + Or; high-fat diet + orlistat 10 mg/kg/day; Nrf2, nuclear factor erythroid 2-related factor 2.

**Figure 2:**
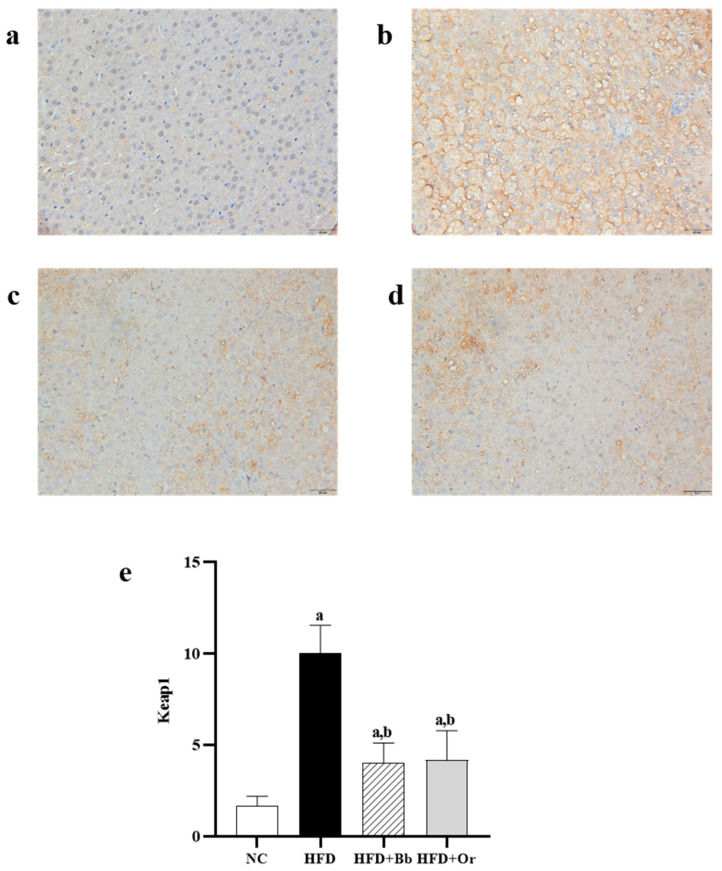
Expressions of Keap1 in liver sections from NC (**a**), HFD (**b**), HFD + Bb (**c**) and HFD + Or (**d**) groups. Magnification, ×400, scale bars represent 20 µm. The staining intensity of Keap1 was quantified (**e**). Values are expressed as mean ± SD, *n* = 7/group. One-way ANOVA, followed by Tukey post-hoc test. ^a^ *p* < 0.05 vs. NC group, ^b^ *p* < 0.05 vs. HFD group. NC, normal control; HFD, high-fat diet; HFD + Bb, high-fat diet + bee bread 0.5 mg/kg/day; HFD + Or; high-fat diet + orlistat 10 mg/kg/day; Keap1, Kelch-like ECH-associated protein 1.

**Figure 3:**
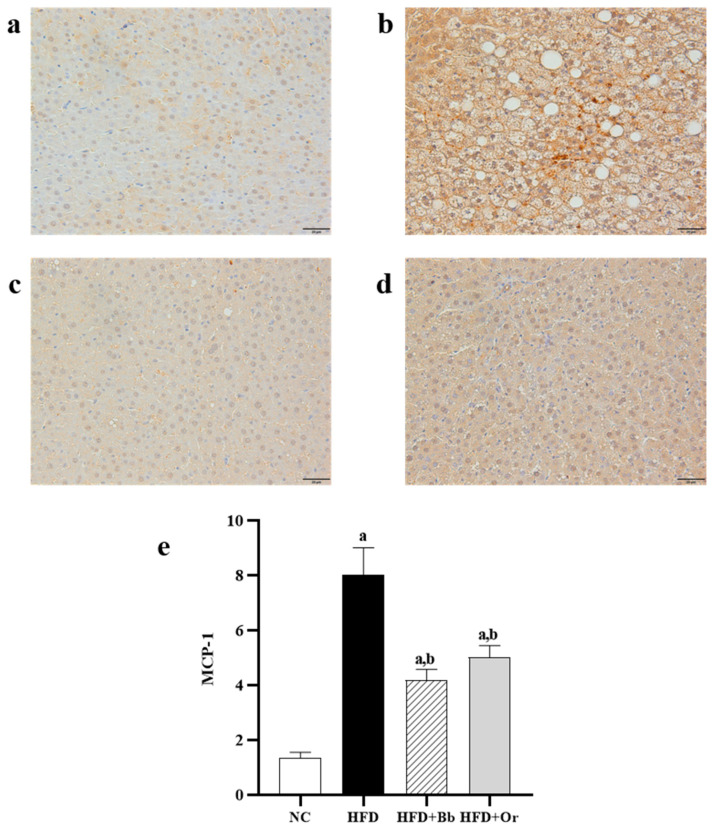
Expressions of MCP-1 in liver sections from NC (**a**), HFD (**b**), HFD + Bb (**c**) and HFD + Or (**d**) groups. Magnification, ×400, scale bars represent 20 µm. The staining intensity of MCP-1 was quantified (**e**). Values are expressed as mean ± SD, *n* = 7/group. One-way ANOVA, followed by Tukey post-hoc test. ^a^ *p* < 0.05 vs. NC group, ^b^ *p* < 0.05 vs. HFD group. NC, normal control; HFD, high-fat diet; HFD + Bb, high-fat diet + bee bread 0.5 mg/kg/day; HFD + Or; high-fat diet + orlistat 10 mg/kg/day; MCP-1, monocyte chemoattractant protein-1.

The translocation of cytoplasmic nuclear factor erythroid 2-related factor 2 (Nrf2) into the nucleus is important for the transcription of antioxidant enzymes. To further explore the effects of bee bread on this antioxidant regulator, the immunohistochemistry localization for Nrf2 protein expression in the liver tissues was determined in this study. In Figure 1a–f, the results demonstrated that the expression of Nrf2 was predominantly found in the cytoplasm of the NC group, whereas the expression of Nrf2 in the cytoplasm was lower and the expression of Nrf2 in the nuclei was higher in the HFD group compared to the NC group in the nuclei. On the contrary, the expression of Nrf2 in the cytoplasm was lower and the expression of Nrf2 in the nucleus was higher in the bee bread group than those in the HFD group. These results indicate increased translocation of cytoplasmic Nrf2 into the nucleus after the intake of bee bread. The findings were also found to be similar in the orlistat group. Moreover, this study also assessed the effects of bee bread on the Nrf2 inhibitor Keap1 protein expression, largely localized in the cytoplasm. High expression of Keap1 protein was observed in the cytoplasm of the HFD group when compared to the NC group. In contrast, the expression of Keap1 protein was reduced after bee bread administration in comparison with the HFD group, which may indicate that bee bread is able to inhibit Keap1 in the liver tissue. A similar result can also be observed in rats administered with orlistat in the orlistat group (Figure 2a–e). We next analyzed hepatic MCP-1 protein levels in the liver tissue, mainly localized in the cytoplasm. IHC analysis exhibited a significant increase in MCP-1 staining in the cytoplasm of the HFD group compared to the NC group. Conversely, the MCP-l level was significantly reduced in the bee bread group in comparison with the HFD group. A similar result was also observed in the orlistat group (Figure 3a–e).

### 3.8. Effects of Bee Bread on NASH Scoring and Fibrosis

**Figure 4:**
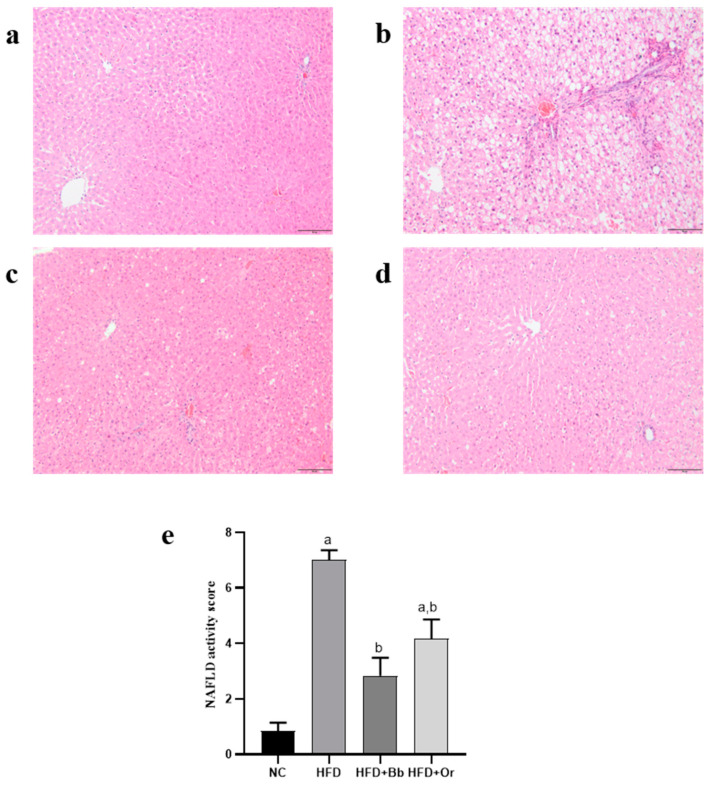
Effects of bee bread on histopathological features of the liver sections from NC (**a**), HFD (**b**), HFD + Bb (**c**) and HFD + Or (**d**) groups using hematoxylin and eosin staining. Magnification, ×200, scale bars represent 50 µm. NASH scoring in all groups (**e**). Values are expressed as mean ± SD, *n* = 7/group. One-way ANOVA, followed by Tukey post-hoc test. ^a^ *p* < 0.05 vs. NC group, ^b^ *p* < 0.05 vs. HFD group. NC, normal control; HFD, high-fat diet; HFD + Bb, high-fat diet + bee bread 0.5 mg/kg/day; HFD + Or; high-fat diet + orlistat 10 mg/kg/day.

**Figure 5:**
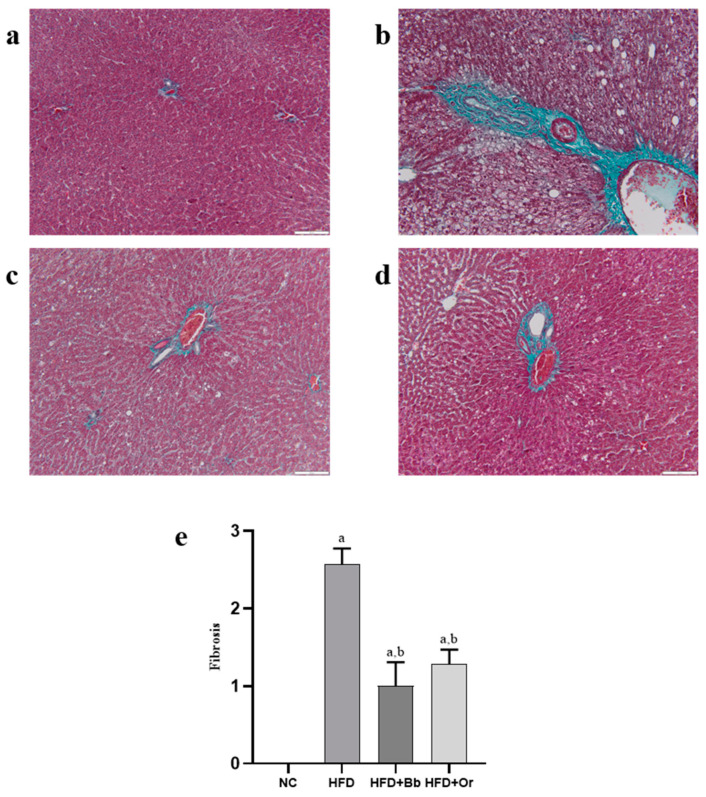
Effects of bee bread on hepatic fibrosis in liver sections from NC (**a**), HFD (**b**), HFD + Bb (**c**) and HFD + Or (**d**) groups assessed using Masson’s Trichrome staining. Magnification, ×200, scale bars represent 100 µm. Fibrosis score in all groups (**e**). Values are expressed as mean ± SD, *n* = 7/group. One-way ANOVA, followed by Tukey post-hoc test. ^a^ *p* < 0.05 vs. NC group, ^b^ *p* < 0.05 vs. HFD group. NC, normal control; HFD, high-fat diet; HFD + Bb, high-fat diet + bee bread 0.5 mg/kg/day; HFD + Or; high-fat diet + orlistat 10 mg/kg/day.

It was observed that the liver sections from the NC group demonstrated normal hepatic architecture and distribution of collagen fibers (stained with green color) (Figure 4a and Figure 5a). In contrast, the HFD group displayed degenerative hepatocytes, steatosis, hepatocyte hypertrophy, inflammatory cell infiltration and marked increases in collagen fibers, periportal fibrosis and bridging fibrosis (portal-central) (Figure 4b and Figure 5b), whereas the liver of rats that received bee bread showed improved liver histology with a mild distribution of collagen fibers around the portal tract, indicating less fibrosis in this group. The effects of the bee bread can be compared with those of orlistat (Figure 4c,d and Figure 5c,d). Likewise, NAS analysis showed no signs of MAFLD observed in the liver of the NC group. On the contrary, the results showed that all the rats that received HFD demonstrated a positive NAS. Nevertheless, the average NAS for the bee bread and orlistat groups reached a maximum of 4 and, therefore, indicated the presence of simple steatosis. Meanwhile, the average NAS for the HFD group was 7, clearly demonstrating the presence of active NASH (Figure 4e). The grading for fibrosis in all the liver sections revealed a significant increase in the fibrosis score in the HFD group relative to the NC group, whereas the bee bread and orlistat groups demonstrated a marked decrease in the fibrosis score in comparison with the HFD group (Figure 5e).

## 4. Discussion

MAFLD is a metabolic disorder associated with obesity, hyperlipidemia, hyperglycemia, insulin resistance, oxidative stress and inflammation [61]. There are now growing evidences that have claimed the health benefits of bee bread in reducing the risk of metabolic syndrome [38,41,42,43,44,45]. Consequently, this study was performed to investigate, for the first time, the possible antioxidative, anti-inflammatory, anti-steatotic and anti-fibrotic effects of bee bread in the HFD-induced MAFLD rat model. This study highlights that bee bread could serve as a potential nutraceutical and functional food that can be used as a treatment for MAFLD. Compared with the NC group, the HFD group showed a number of MAFLD-related metabolic disorders, including obesity, hyperlipidemia, liver dysfunction, redox state imbalance and inflammation. The daily administration of bee bread (0.5 g/kg/day) for 12 weeks effectively ameliorated obesity and improved insulin resistance and hyperlipidemia in the rats. Furthermore, bee bread reduced hepatic steatosis and protected the rats from progression into NASH.

Obesity has become the major contributor to the prevalence of MAFLD worldwide. Hence, obesity is one of the predisposing factors for the progression of MAFLD, which is in agreement with our findings [62]. Our study showed that an excessive ingestion of HFD for 12 weeks significantly increased the obesity parameters such as body weight gain, BMI and AC/TC ratio in the HFD group compared with those in the NC group. This could be attributed to the greater component of fat in the HFD administration, which comprised of 31% fat in comparison with only 12% fat in the standard diet [41]. Moreover, excess dietary fat ingested by an individual corresponds to more accumulation of fat mass in the abdominal and visceral areas [63]. The present study demonstrated that bee bread could repress HFD-induced obesity, which was presented by a marked reduction in the obesity parameters after 12 weeks of administration. This is in agreement with a previously reported study [41]. The beneficial effects of bee bread presented in this study could be attributed to its rich source of phenolic compounds including hydroxycinnamic acid derivatives, caffeic acid, gallic acid, ferulic acid, quercetin, apigenin, kaempferol and mangiferin [64], which have lipid-lowering and anti-obesity effects, as reported in previous studies [41,65,66].

Parallel to the change in body weight, we also observed a substantial increase in adipose tissue weight in the HFD group compared with those in the NC group. Numerous data from previous studies reported the role of adipose tissue in regulating metabolic activities of the brain, muscle and cardiovascular system [67]. The adipocytokines secreted by the adipocytes including adiponectin, leptin, TNF-α, resistin and plasminogen activator 1 (PAI-1) control appetite, insulin sensitivity and inflammation; hence, they play their role in the MAFLD’s pathogenesis and its development to NASH [25]. An elevation in adiponectin level has been connected with the inhibitions of hepatic lipid accumulation and insulin resistance as well as exerting its hepatoprotective actions by reducing the production of pro-inflammatory cytokines and increasing the expressions of anti-inflammatory IL-10 and IL-1 receptor antagonists [68,69]. This study also demonstrated a marked decrease in the level of serum adiponectin in the HFD group compared with the NC group. Thus, HFD might interrupt the function of adipose tissue, which in turn results in insulin resistance and obesity-related metabolic diseases [67]. In contrast, the administration of bee bread for 12 weeks markedly elevated the level of adiponectin compared with the HFD group, indicating that bee bread might have the potential to improve the adipose tissue dysfunction.

An increase in serum liver enzymes is identified as the first manifestation of liver disease [70]. In order to confirm the hepatoprotective effects of bee bread against liver injury, liver function tests were evaluated in this study. The present study demonstrated the elevation of liver enzymes, notably ALT, AST and ALP, in the HFD group, as compared with the NC group, which is in line with a previous study [71]. The intake of bee bread significantly reduced the levels of liver enzymes, which is in line with previously reported studies in different models; streptozotocin-induced diabetic rats [72], aluminum-induced hepatotoxicity rats [39] and alcohol-induced liver disease patients [38].

These positive findings were further confirmed by histopathological examination of liver sections obtained from all animal groups. The liver from the HFD group showed degenerative hepatocytes, steatosis, hepatocyte hypertrophy, inflammatory cell infiltration and the presence of active NASH, together with marked increases in collagen fiber as well as periportal fibrosis and bridging fibrosis (portal-central). Hepatic fibrosis is initiated by hepatic lipotoxicity due to excessive hepatic lipid accumulation and results in chronic liver injury, inflammation and activation of hepatic stellate cells leading to excessive buildup of extracellular matrix proteins, primarily collagen in the liver tissue [73]. It is recognized as one of the main features of MAFLD and if left untreated, MAFLD can further progress into cirrhosis and hepatocellular carcinoma [1]. The administration of bee bread markedly ameliorated these negative changes. Notably, bee bread has the ability to improve and prevent most of the features of MAFLD, from histological changes to fibrosis, hence, providing some hepatoprotection against MAFLD.

Indeed, type 2 diabetes has been reported to share a link with hyperlipidemia and is recognized as a co-morbidity commonly established in obese patients [74]. The Increased consumption of an HFD resulted in an increased peripheral insulin resistance, which was demonstrated by the increased serum fasting glucose, hyperinsulinemia and increased HOMA-IR in the HFD group. Bee bread administration significantly alleviated the levels of serum fasting glucose, insulin and HOMA-IR, thus suggesting that it has an insulin-sensitizing effect. This might be ascribed to the up-regulated levels of glucose transporters GLUT1 and GLUT3 as demonstrated in the testes of obese male rats after intervention with bee bread [42]. Chronic HFD feeding is also responsible for the increases in blood lipid levels [75]. This study showed increased concentrations of serum TG, TC and LDL and reduced concentration of serum HDL in the HFD group than those in the NC group. Bee bread improved hyperlipidemia by lowering TG, TC and LDL levels, and, in contrast, increased the HDL level. Our results coincide with previous studies that reported the hypolipidemic effects of bee bread [41,42,76]. This beneficial property of bee bread could be ascribed to the presence of flavonoids, which have been reported to exert their antioxidative property towards LDL by inhibiting its susceptibility to oxidation [77]. Moreover, the interaction between HDL-associated enzyme paraoxonase-1 (PON1) with the flavonoids has been reported to be responsible for many of HDL’s antioxidative properties, which might elucidate the reduced levels of oxidative stress makers in the present study [78]. Hence, evaluating the level of PON1 in the serum and liver of the HFD-induced MAFLD model is warranted in future studies. In addition, a previous study reported the ability of saponin in bee bread to reduce non-HDL lipids concentration in the blood by its interaction with dietary fat constituents, and, subsequently, increase the excretion of lipid in feces [79].

Hepatic lipid buildup is recognized as the “first hit” theory to describe the pathogenesis of MAFLD and, subsequently, the “second hit” theory, which consists of inflammatory cytokines, adipokines, mitochondrial dysfunction and oxidative stress [80]. Imbalance in hepatic lipid metabolism including a disturbance in fatty acid uptake, de novo lipogenesis (DNL), lipolysis and fatty acid oxidation results in abnormal lipid deposition in the hepatocytes [81]. The present study demonstrated increased hepatic levels of TG and TC and, concomitantly, increases in the absolute liver weight and liver index of the HFD group. In contrast, the intake of bee bread reduced these hepatic lipid contents, liver weights and hepatic steatosis in the bee bread group. In addition, insulin resistance is strongly related to hepatic lipid accumulation [82] in which peripheral insulin resistance enhances lipolysis and free fatty acid uptake into the liver tissue, hence, leading to hepatic lipid accumulation [83]. Furthermore, insulin resistance regulates hepatic DNL via its action on sterol regulatory element binding protein-1c (SREBP-1c) and carbohydrate response element binding protein (ChREBP), the main transcription factors, which are essential to regulate the expression of genes involved in DNL and lipid synthesis in the liver [81]. Hence, reduced hepatic lipid accumulation and insulin resistance after bee bread administration in the present study might be ascribed to its inhibition effect on hepatic genes-related to DNL as reported by a previous study [45].

The liver is known as a powerhouse organ that performs various crucial tasks, ranging from the production of proteins, cholesterol and bile to storing vitamins, minerals and even carbohydrates, as well as breaking down toxins such as alcohol, medications and natural by-products of metabolism to maintain metabolic homeostasis in an organism. Hence, it is mostly susceptible to oxidative stress [84,85], which, in turn, is incriminated in the pathogenesis of MAFLD [17]. Our present study proved that an HFD stimulated oxidative stress with a marked reduction in the activities of antioxidant enzymes. This is in line with a few previous findings that reported that there were elevations in the levels of free radicals in the rat model of MAFLD, meanwhile, the activities of antioxidant enzymes were reduced [86,87]. The present study reported the hepatoprotective role of bee bread on oxidative stress in HFD-induced MAFLD rats in which the intake of bee bread (0.5 g/kg/day) for 12 weeks significantly reduced the levels of oxidative stress markers (TBARS and NO), meanwhile, it increased the activities of enzymatic antioxidants (GPx, GST, GR, SOD and CAT) and GSH level. It is well-known that the transcription factor Nrf2 plays a critical role in the cellular defense mechanism against MAFLD-induced liver oxidative stress by down-regulating the genes responsible for hepatic lipid accumulation in rats, thereby preventing further damage exerted by lipids in the hepatocytes [88]. It is reported that Keap1 deficiency prevents ethanol-induced ROS overproduction, while Nrf2 knockout triggers ROS up-regulation in mouse primary hepatocytes [89]. In the present study, we reported the increased translocation of cytoplasmic Nrf2 into the nucleus of the HFD group compared to the NC group. However, the analyses of antioxidant enzymes activities showed a significant reduction in these enzymes GPx, GST, GR, SOD and CAT activities in the HFD group compared with those in the NC group. Although there was an elevation in Nrf2 expression in the nucleus of the HFD group, the decreased antioxidant enzymes’ activity might be due to deactivation or a reduction in enzyme synthesis caused by the overproduction of ROS, as demonstrated by high levels of TBARS and NO in this group [90]. Apart from that, bee bread enhanced the translocation of Nrf2 from the cytoplasm into the nucleus, as demonstrated by the higher expression of nuclear Nrf2 in comparison with cytoplasmic Nrf2 following bee bread administration, compared to the HFD group. These results might also support our findings on decreased oxidative stress levels and increased antioxidant enzymes’ activity in the bee bread group as compared to the HFD group, probably by enhancing the synthesis of these enzymes. Hence, evaluating their mRNA levels in future study is warranted to further validate the action of bee bread on these enzymes’ syntheses. Similarly, the significantly decreased Keap1 expression following bee bread administration indicated that bee bread might suppress Keap1 to promote Nrf2 translocation and, in turn, might up-regulate the activities of antioxidant enzymes in the HFD-fed rat livers, thus decreasing oxidative stress [88]. In addition to immunochemistry analysis, it is also recommended to further corroborate the levels of cytoplasmic Nrf2 and Keap1, and nuclear Nrf2 using the Western blot technique and mRNA analysis in future studies.

As mentioned above, defective lipid metabolism leads to hepatic lipid overload, which results in lipid peroxidation, leading to oxidative stress, inflammation and fibrosis. Excessive production of ROS stimulates the hepatocytes to secrete more cytokine, subsequently leading to liver damage [91]. Elevated hepatic lipid has been linked with activated NF-κβ and increased productions of TNF-α, IL-1 and Il-6, while suppression of NF-κβ in the liver down-regulates the expression of genes encoding these pro-inflammatory mediators [92]. TNF-α is responsible for a range of intracellular signaling systems, including the activation of NF-κβ, and has been linked with the increased activity of Jun N-terminal kinase, which is essential in promoting hepatic insulin resistance [93]. It is also reported that TNF-α is involved in hepatic fatty acid synthesis, increases serum triglyceride level, activates the production of VLDL from the liver and stimulates both hepatocytes’ cell death and proliferation, thus, is crucially involved in the pathogenesis of liver fibrosis [94]. This is confirmed by our present study in which significantly increased levels of pro-inflammatory mediators TNF-α and NF-κβ, and a significantly decreased level of anti-inflammatory cytokine IL-10 were observed in the liver of the HFD group, as well as elevation in serum lipids and the deposition of collagen fibers, which indicated the presence of liver fibrosis in this group. Bee bread (0.5 g/kg/day for 12 weeks) has been previously reported to suppress TNF-α and NF-κβ, and increase IL-10 levels in obesity-induced aortic vascular damage [76] and HFD-induced renal damage [43] rats. Our present findings are in agreement with previous reported studies, and further support the beneficial properties of bee bread against inflammation and fibrosis. MCP-1, also known as CCL2, is a prototypical inflammatory chemokine secreted by hepatic stellate cells upon tissue injury [95]. Previous studies have linked increased hepatic expression of MCP-1 with hepatic steatosis and insulin resistance in HFD-fed rats [96,97], as well as in patients with advanced liver fibrosis [98]. Similarly, the present study demonstrated a significantly increased hepatic MCP-1 expression in the HFD group compared with those in the NC group, further indicating the progression of hepatic steatosis towards NASH and fibrosis, as well as the increased inability of the liver to manage fat infiltration. Furthermore, an increased level of MCP-1 is reported to be stimulated by an increased level of TNF-α in hepatocytes [69], which was also demonstrated in the HFD group in the present study. Conversely, the intake of bee bread markedly reduced the expression of MCP-1 in the liver tissue of HFD-fed rats, suggesting the anti-inflammatory property of bee bread.

## 5. Conclusions

The present study demonstrated that administration of bee bread at 0.5 g/kg/day for 12 weeks provided hepatoprotection against MAFLD in rats. Bee bread significantly reduced obesity, hyperlipidemia, liver injury, hyperglycemia, insulin resistance, hepatic steatosis and fibrosis, by exerting its antioxidant, anti-inflammatory, anti-steatotic and anti-fibrotic effects in the liver, which are beneficial in protecting against NASH and advanced fibrosis.

## Figures and Tables

**Table 1:** Anthropometrical and nutritional parameters of rats in the experimental groups.

	NC	HFD	HFD + Bb	HFD + Or
Body weight gain (g)	99.57 ± 26.26	208.40 ± 31.54 ^a^	114.00 ± 9.27 ^b^	162.30 ± 17.05 ^a,b,c^
BMI (g/cm2)	0.66 ± 0.02	0.85 ± 0.05 ^a^	0.65 ± 0.05 ^b^	0.73 ± 0.06 ^b,c^
AC/TC ratio	0.95 ± 0.09	1.13 ± 0.03 ^a^	0.98 ± 0.07 ^b^	1.01 ± 0.11

Values are expressed as mean ± SD, *n* = 7/group. NC, normal control; HFD, high-fat diet; HFD + Bb, high-fat diet + bee bread 0.5 g/kg/day; HFD + Or, high-fat diet + orlistat 10 mg/kg/day; BMI, body mass index; AC, abdominal circumference; TC, thoracic circumference. One-way ANOVA, followed by Tukey post-hoc test. ^a^ *p* < 0.05 vs. NC group, ^b^ *p* < 0.05 vs. HFD group, ^c^ *p* < 0.05 vs. HFD + Bb group.

**Table 2:** Serum biochemical parameters of rats in the experimental groups.

	NC	HFD	HFD + Bb	HFD + Or
TG (mmol/L)	0.48 ± 0.07	0.93 ± 0.09 ^a^	0.56 ± 0.08 ^b^	0.75 ± 0.16 ^a,b,c^
TC (mmol/L)	1.61 ± 0.16	2.52 ± 0.66 ^a^	1.92 ± 00.19 ^b^	1.84 ± 0.19 ^b^
LDL (mmol/L)	0.89 ± 0.17	1.70 ± 0.59 ^a^	1.04 ± 0.19 ^b^	0.91 ± 0.20 ^b^
HDL (mmol/L)	0.53 ± 0.05	0.40 ± 0.06 ^a^	0.60 ± 0.03 ^b^	0.61 ± 0.09 ^b^
ALT (U/L)	48.43 ± 4.50	70.14 ± 8.75 ^a^	52.00 ± 5.60 ^b^	54.43 ± 7.87 ^b^
AST (U/L)	113.00 ± 11.94	149.10 ± 41.92 ^a^	91.00 ± 19.45 ^b^	94.71 ± 10.50 ^b^
ALP (U/L)	126.20 ± 22.97	361.20 ± 41.34 ^a^	204.30 ± 44.42 ^b^	247.70 ± 24.38 ^a,b^
Adiponectin (ng/mL)	3.79 ± 0.60	2.10 ± 0.33 ^a^	3.31 ± 0.35 ^b^	3.53 ± 0.40 ^b^

Values are expressed as mean ± SD, *n* = 7/group. NC, normal control; HFD, high-fat diet; HFD + Bb, high-fat diet + bee bread 0.5 g/kg/day; HFD + Or, high-fat diet + orlistat 10 mg/kg/day; TG, triglyceride; TC, total cholesterol; LDL, low-density lipoprotein; HDL, high-density lipoprotein; ALT, alanine aminotransferase; AST, aspartate aminotransferase; ALP, alkaline phosphatase. One-way ANOVA, followed by Tukey post-hoc test. ^a^ *p* < 0.05 vs. NC group, ^b^ *p* < 0.05 vs. HFD group, ^c^ *p* < 0.05 vs. HFD + Bb group.

**Table 3:** Serum glucose and insulin resistance of rats in the experimental groups.

	NC	HFD	HFD + Bb	HFD + Or
Glucose (mg/dL)	65.00 ± 4.56	85.20 ± 10.57 ^a^	66.20 ± 4.21 ^b^	71.80 ± 7.29 ^b^
Insulin (ng/mL)	0.61 ± 0.20	2.56 ± 1.75 ^a^	0.50 ± 0.19 ^b^	0.58 ± 0.27 ^b^
HOMA-IR	0.10 ± 0.03	0.33 ± 0.08 ^a^	0.12 ± 0.01 ^b^	0.14 ± 0.04 ^b^

Values are expressed as mean ± SD, *n* = 7/group. NC, normal control; HFD, high-fat diet; HFD + Bb, high-fat diet + bee bread 0.5 g/kg/day; HFD + Or, high-fat diet + orlistat 10 mg/kg/day; HOMA-IR, homeostatic model of assessment-insulin resistance. One-way ANOVA, followed by Tukey post-hoc test. ^a^ *p* < 0.05 vs. NC group, ^b^ *p* < 0.05 vs. HFD group.

**Table 4:** Absolute liver weight, liver index and liver lipid content of rats in the experimental groups.

	NC	HFD	HFD + Bb	HFD + Or
Epididymal adipose tissue weight (g)	2.97 ± 0.38	11.59 ± 3.18 ^a^	4.16 ± 0.59 ^b^	5.91 ± 2.1 ^a,b^
Peritoneal adipose tissue weight (g)	2.67 ± 0.89	14.14 ± 6.01 ^a^	5.62 ± 3.12 _b_	6.59 ± 2.86 ^b^
Perirenal adipose tissue weight (g)	0.21 ± 0.08	0.61 ± 0.12 ^a^	0.31 ± 0.13 ^b^	0.39 ± 0.03 ^b^
Total adipose tissue weight (g)	6.57 ± 0.93	26.78 ± 6.65 ^a^	7.89 ±1.72 ^b^	9.40 ± 1.50 ^b^
Absolute liver weight (g)	8.92 ± 0.83	15.38 ± 1.57 ^a^	11.12 ± 1.15 ^a,b^	13.49 ± 1.56 ^a,c^
Liver index (%)	2.45 ± 0.08	3.41 ± 0.21 ^a^	3.00 ± 0.09 ^a,b^	3.17 ± 0.28 ^a^
Liver TG (ng/g tissue)	18.36 ± 3.65	36.75 ± 5.59 ^a^	23.93 ± 2.79 ^b^	24.18 ± 3.15 ^b^
Liver TC (ng/g tissue)	15.54 ± 3.84	32.31 ± 7.20 ^a^	16.51 ± 2.80 ^b^	21.98 ± 1.12 ^b^

Values are expressed as mean ± SD, *n* = 7/group. NC, normal control; HFD, high-fat diet; HFD + Bb, high-fat diet + bee bread 0.5 g/kg/day; HFD + Or, high-fat diet + orlistat 10 mg/kg/day; TG, triglyceride; TC, total cholesterol. One-way ANOVA, followed by Tukey post-hoc test. ^a^ *p* < 0.05 vs. NC group, ^b^ *p* < 0.05 vs. HFD group, ^c^ *p* < 0.05 vs. HFD + Bb group.

**Table 5:** Oxidative stress status of rats in the experimental groups.

	NC	HFD	HFD + Bb	HFD + Or
TBARS (nmol/mg protein)	2.45 ± 0.17	6.25 ± 1.49 ^a^	2.18 ± 0.37 ^b^	2.07 ± 0.66 ^b^
NO (µmol/g protein)	0.82 ± 0.06	1.13 ± 0.12 ^a^	0.83 ± 0.09 ^b^	0.96 ± 0.06 ^b^
GPx (unit/mg protein)	31.56 ± 3.76	14.93 ± 3.22 ^a^	29.24 ± 5.48 ^b^	27.44 ± 2.56 ^b^
GST (unit/mg protein)	15.09 ± 1.70	5.37 ± 2.05 ^a^	12.13 ± 2.84 ^b^	11.35 ± 2.59 ^b^
GSH (nmol/mg protein)	4.63 ± 0.36	2.20 ± 0.73 ^a^	3.75 ± 0.55 ^b^	3.67 ± 0.56 ^b^
GR (unit/mg protein)	17.66 ± 3.15	11.52 ± 2.35 ^a^	16.48 ± 2.36 ^b^	16.44 ± 2.48 ^b^
SOD (unit/mg protein)	6.06 ± 0.60	1.35 ± 0.80 ^a^	5.78 ± 0.41 ^b^	5.13 ± 0.18 ^b^
CAT (unit/mg protein)	26.48 ± 5.46	13.70 ± 1.95 ^a^	25.50 ± 2.89 ^b^	21.35 ± 4.38 ^b^

Values are expressed as mean ± SD, *n* = 7/group. NC, normal control; HFD, high-fat diet; HFD + Bb, high-fat diet + bee bread 0.5 g/kg/day; HFD + Or, high-fat diet + orlistat 10 mg/kg/day; TBARS, thiobarbituric acid reactive substances; NO, nitric oxide; GPx, glutathione peroxidase; GST, glutathione S-transferase; GSH, glutathione; GR, glutathione reductase SOD; superoxide dismutase; CAT; catalase. One-way ANOVA, followed by Tukey post-hoc test. ^a^ *p* < 0.05 vs. NC group, ^b^ *p* < 0.05 vs. HFD group.

**Table 6:** Liver inflammatory markers of rats in the experimental groups.

	NC	HFD	HFD + Bb	HFD + Or
TNF-α (ng/mg protein)	12.42 ± 0.99	19.65 ± 5.15 ^a^	13.68 ± 1.37 ^b^	14.46 ± 1.23 ^b^
NF-κβ (ng/g protein)	20.43 ± 7.95	68.29 ± 14.27 ^a^	31.29 ± 4.93 ^b^	32.70 ± 6.97 ^b^
IL-10 (ng/g protein)	58.31 ± 5.98	26.43 ± 8.69 ^a^	65.07 ± 11.81 ^b^	64.67 ± 7.69 ^b^

Values are expressed as mean ± SD, *n* = 7/group. NC, normal control; HFD, high-fat diet; HFD + Bb, high-fat diet + bee bread 0.5 g/kg/day; HFD + Or, high-fat diet + orlistat 10 mg/kg/day; TNF-α; tumor necrosis factor alpha; NF-κβ; nuclear factor kappa β; IL-10; interleukin 10. One-way ANOVA, followed by Tukey post-hoc test. ^a^ *p* < 0.05 vs. NC group, ^b^ *p* < 0.05 vs. HFD group.

## Data Availability

All of the data is contained within the article.

## References

[1] Perumpail B.J., Khan M.A., Yoo E.R., Cholankeril G., Kim D., Ahmed A. (2017). Clinical epidemiology and disease burden of nonalcoholic fatty liver disease. World J. Gastroenterol..

[2] Araújo A.R., Rosso N., Bedogni G., Tiribelli C., Bellentani S. (2018). Global epidemiology of non-alcoholic fatty liver disease/non-alcoholic steatohepatitis: What we need in the future. Liver Int..

[3] O’Shea R.S., Dasarathy S., McCullough A.J., Practice Guideline Committee of the American Association for the Study of Liver Diseases, Practice Parameters Committee of the American College of Gastroenterology (2010). Alcoholic liver disease. Hepatology.

[4] Golabi P., Paik J.M., Arshad T., Younossi Y., Mishra A., Younossi Z.M. (2020). Mortality of NAFLD according to the body composition and presence of metabolic abnormalities. Hepatol. Commun..

[5] Younossi Z.M., Koenig A.B., Abdelatif D., Fazel Y., Henry L., Wymer M. (2016). Global epidemiology of nonalcoholic fatty liver disease-Meta-analytic assessment of prevalence, incidence, and outcomes. Hepatology.

[6] Younossi Z.M., Golabi P., de Avila L., Paik J.M., Srishord M., Fukui N., Qiu Y., Burns L., Afendy A., Nader F. (2019). The global epidemiology of NAFLD and NASH in patients with type 2 diabetes: A systematic review and meta-analysis. J. Hepatol..

[7] Tanase D.M., Gosav E.M., Costea C.F., Ciocoiu M., Lacatusu C.M., Maranduca M.A., Ouatu A., Floria M. (2020). The intricate relationship between type 2 Diabetes Mellitus (T2DM), Insulin Resistance (IR) and Nonalcoholic Fatty Liver Disease (NAFLD). J. Diabetes Res..

[8] Coccia F., Testa M., Guarisco G., Di Cristofano C., Silecchia G., Leonetti F., Gastaldelli A., Capoccia D. (2020). Insulin resistance, but not insulin response, during oral glucose tolerance test (OGTT) is associated to worse histological outcome in obese NAFLD. Nutr. Metab. Cardiovas..

[9] Chen W., Shao S., Cai H., Han J., Guo T., Fu Y., Yu C., Zhao M., Bo T., Yao Z. (2020). Comparison of erythrocyte membrane lipid profiles between NAFLD patients with or without hyperlipidemia. Int. J. Endocrinol..

[10] Zhao Y.-C., Zhao G.-J., Chen Z., She Z.-G., Cai J., Li H. (2020). Nonalcoholic Fatty Liver Disease: An emerging driver of hypertension. Hypertens. AHA.

[11] Eslam M., Newsome P.N., Sarin S.K., Anstee Q.M., Targher G., Romer-Gomez M., Zelber-Sagi S., Wai-Sun Wong V., Dufor J.F., Schattenberg J.M. (2020). A new definition for metabolic dysfunction-associated fatty liver disease: An international consensus statement. J. Hepatol..

[12] Eslam M., Sanyal A.J., George J. (2020). MAFLD: A consensus-driven proposed nomenclature for metabolic associated fatty liver disease. Gastroenterology.

[13] Buzzetti E., Pinzani M., Tsochatzis E.A. (2016). The multiple-hit pathogenesis of non-alcoholic fatty liver disease (NAFLD). Metabolism.

[14] Zhang Q., Yu K., Cao Y., Luo Y., Liu Y., Zhao C. (2021). Mir-125b promotes the nf-kappab-mediated inflammatory response in nafld via directly targeting tnfaip3. Life Sci..

[15] Rahman M.M., Alam M.N., Ulla A., Sumi F.A., Subhan N., Khan T., Sikder B., Hossain H., Reza H.M., Alam M.A. (2017). Cardamom powder supplementation prevents obesity, improves glucose intolerance, inflammation and oxidative stress in liver of high carbohydrate high fat diet induced obese rats. Lipids Health Dis..

[16] Hwang I., Uddin M.J., Pak E.S., Kang H., Jin E.J., Jo S., Kang D., Lee H., Ha H. (2020). The impaired redox balance in peroxisomes of catalase knockout mice accelerates nonalcoholic fatty liver disease through endoplasmic reticulum stress. Free Radic. Biol. Med..

[17] Chambel S.S., Santos-Gonçalves A., Duarte T.L. (2015). The Dual Role of Nrf2 in Nonalcoholic Fatty Liver Disease: Regulation of Antioxidant Defenses and Hepatic Lipid Metabolism. BioMed Res. Int..

[18] Okada K., Warabi E., Sugimoto H., Horie M., Gotoh N., Tokushige K., Hashimoto E., Utsunomiya H., Takahashi H., Ishii T. (2013). Deletion of Nrf2 leads to rapid progression of steatohepatitis in mice fed atherogenic plus high-fat diet. J. Gastroenterol..

[19] Liu X., Zhang X., Ding Y., Zhou W., Tao L., Lu P., Wang Y., Hu R. (2017). Nuclear Factor E2-Related Factor-2 Negatively Regulates NLRP3 Inflammasome Activity by Inhibiting Reactive Oxygen Species-Induced NLRP3 Priming. Antioxid. Redox Signal..

[20] Kensler T.W., Wakabayashi N., Biswal S. (2007). Cell survival responses to environmental stresses via the Keap1-Nrf2-ARE pathway. Annu. Rev. Pharmacol. Toxicol..

[21] Tilg H., Moschen A.R. (2010). Evolution of inflammation in nonalcoholic fatty liver disease: The multiple parallel hits hypothesis. Hepatology.

[22] Del Campo J.A., Gallego P., Grande L. (2018). Role of inflammatory response in liver diseases: Therapeutic strategies. World J. Hepatol..

[23] Van Herck M.A., Weyler J., Kwanten W.J., Dirinck E.L., De Winter B.Y., Francque S.M., Vonghia L. (2019). The Differential Roles of T Cells in Non-alcoholic Fatty Liver Disease and Obesity. Front. Immunol..

[24] Nallagangula K.S., Nagaraj S.K., Venkataswamy L., Chandrappa M. (2018). Liver fibrosis: A compilation on the biomarkers status and their significance during disease progression. Future Sci. OA.

[25] Gatselis N.K., Ntaios G., Makaritsis K., Dalekos G.N. (2014). Adiponectin: A key playmaker adipocytokine in non-alcoholic fatty liver disease. Clin. Exp. Med..

[26] Yang Z., Zhu M.Z., Zhang Y.B., Wen B.B., An H.M., Ou X.C., Xiong Y.F., Lin H.Y., Liu Z.H., Huang J.A. (2019). Coadministration of epigallocatechin-3-gallate (EGCG) and caffeine in low dose ameliorates obesity and nonalcoholic fatty liver disease in obese rats. Phytother. Res..

[27] Adams L.A., Anstee Q.M., Tilg H., Targher G. (2017). Non-alcoholic fatty liver disease and its relationship with cardiovascular disease and other extrahepatic diseases. Gut.

[28] Sumida Y., Yoneda M. (2018). Current and future pharmacological therapies for NAFLD/NASH. J. Gastroenterol..

[29] Barajas J., Cortes-Rodriguez M., Rodríguez-Sandoval E. (2012). Effect of temperature on the drying process of bee pollen from two zones of colombia. J. Food Process Eng..

[30] Vásquez A., Olofsson T.C. (2009). The Lactic Acid Bacteria Involved in the Production of Bee Pollen and Bee Bread. J. Apic. Res..

[31] Barene I., Daberte I., Siksna S. (2014). Investigation of Bee Bread and Development of Its Dosage Forms. Med. Teor. Prakt..

[32] Anderson K.E., Carroll M.J., Sheehan T.I.M., Mott B.M., Maes P., Corby-Harris V. (2014). Hive-stored pollen of honey bees: Many lines of evidence are consistent with pollen preservation, not nutrient conversion. Mol. Ecol..

[33] Kieliszek M., Piwowarek K., Kot A.M., Błażejak S., Chlebowska-Śmigiel A., Wolska I. (2017). Pollen and bee bread as new health-oriented products: A review. Trends Food Sci. Technol..

[34] Habryka C., Kruczek M., Drygas B. (2016). Bee products used in apitherapy. World Sci. News.

[35] Abouda Z., Zerdani I., Kalalou I., Faid M., Ahami M.T. (2011). The antibacterial activity of Moroccan bee bread and bee-pollen (fresh and dried) against pathogenic bacteria. Res. J. Microbiol..

[36] Baltrušaitytė V., Venskutonis P.R., Čeksterytė V. (2007). Radical scavenging activity of different floral origin honey and beebread phenolic extracts. Food Chem..

[37] Sobral F., Calhelha R.C., Barros L., Dueñas M., Tomás A., Santos-Buelga C., Ferreira I.C. (2017). Flavonoid composition and antitumor activity of bee bread collected in northeast Portugal. Molecules.

[38] Ceksteryte V., Balzekas J. (2012). The use of beebread—Honey mixture in the treatment of liver diseases in alcohol-dependent patients. J. Chem. Technol..

[39] Bakour M., Al-Waili N.S., El Menyiy N., Imtara H., Figuira A.C., Al-Waili T., Lyoussi B. (2017). Antioxidant activity and protective effect of bee bread (honey and pollen) in aluminum-induced anemia, elevation of inflammatory makers and hepato-renal toxicity. J. Food Sci. Technol..

[40] Saral Ö., Yildiz O., Aliyazicioğlu R., Yuluğ E., Canpolat S., Öztürk F., Kolayli S. (2016). Apitherapy products enhance the recovery of CCL_4_-induced hepatic damages in rats. Turk. J. Med. Sci..

[41] Othman Z.A., Ghazali W.S.W., Noordin L., Yusof N.A.M., Mohamed M. (2020). Phenolic Compounds and the Anti-Atherogenic Effect of Bee Bread in High-Fat Diet-Induced Obese Rats. Antioxidants.

[42] Suleiman J.B., Nna V.U., Zakaria Z., Othman Z.A., Eleazu C.O., Bakar A.B.A., Ahmad A., Usman U.Z., Rahman W.F.W.A., Mohamed M. (2020). Protective effects of bee bread on testicular oxidative stress, NF-κB-mediated inflammation, apoptosis and lactate transport decline in obese male rats. Biomed. Pharmacother..

[43] Eleazu C., Suleiman J.B., Othman Z.A., Zakaria Z., Nna V.U., Hussain N.H.N., Mohamed M. (2020). Bee bread attenuates high fat diet induced renal pathology in obese rats via modulation of oxidative stress, downregulation of NF-kB mediated inflammation and Bax signalling. Arch. Physiol. Biochem..

[44] Markiewicz-Żukowska R., Naliwajko S.K., Bartosiuk E., Moskwa J., Isidorov V., Soroczyńska J., Borawska M.H. (2013). Chemical composition and antioxidant activity of beebread, and its influence on the glioblastoma cell line (U87MG). J. Apic. Sci..

[45] Li Z., Huang Q., Liu Y., Peng C., Zeng Z. (2021). Natural bee bread positively regulates lipid metabolism in rats. Int. J. Agric. Sci. Food Technol..

[46] Zaitone S.A., Essawy S. (2012). Addition of a low dose of rimonabant to orlistat therapy decreases weight gain and reduces adiposity in dietary obese rats. Clin. Exp. Pharmacol. Physiol..

[47] Novelli E.L.B., Diniz Y.S., Galhardi C.M., Ebaid G.M.X., Rodrigues H.G., Mani F. (2007). Anthropometrical parameters and markers of obesity in rats. Lab. Anim..

[48] Friedewald W.T., Levy R.I., Fredrickson D.S. (1972). Estimation of the concentration of low-density lipoprotein cholesterol in plasma, without use of the preparative ultracentrifuge. Clin. Chem..

[49] Roza N.A.V., Possignolo L.F., Palanch A.C., Gontijo J.A.R. (2016). Effect of long-term high-fat diet intake on peripheral insulin sensibility, blood pressure, and renal function in female rats. Food Nutr. Res..

[50] Chatterjee P.K., Cuzzocrea S., Brown P.A., Zacharowski K., Stewart K.N., Mota-Filipe H., Thiemermann C. (2000). Tempol, a membrane-permeable radical scavenger, reduces oxidant stress-mediated renal dysfunction and injury in the rat. Kidney Int..

[51] Al Batran R., Al-Bayaty F., Jamil Al-Obaidi M.M., Abdualkader A.M., Hadi H.A., Ali H.M., Abdulla M.A. (2013). In vivo antioxidant and antiulcer activity of *Parkia speciosa* ethanolic leaf extract against ethanol-induced gastric ulcer in rats. PLoS ONE.

[52] Chandran G., Sirajudeen K., Yusoff N., Syamimi N., Swamy M., Samarendra M.S. (2014). Effect of the antihypertensive drug enalapril on oxidative stress markers and antioxidant enzymes in kidney of spontaneously hypertensive rat. Oxid. Med. Cell. Long..

[53] Winiarska K., Focht D., Sierakowski B., Lewandowski K., Orlowska M., Usarek M. (2014). NADPH oxidase inhibitor, apocynin, improves renal glutathione status in Zucker diabetic fatty rats: A comparison with melatonin. Chem. Biol. Interact..

[54] Habig W.H., Pabst M.J., Jakoby W.B. (1974). Glutathione S-transferases: The first enzymatic step in mercapturic acid formation. J. Biol. Chem..

[55] Annuk M., Zilmer M., Lind L., Linde T., Fellström B. (2001). Oxidative stress and endothelial function in chronic renal failure. J. Am. Soc. Nephrol..

[56] Luchese C., Pinton S., Nogueira C.W. (2009). Brain and lungs of rats are differently affected by cigarette smoke exposure: Antioxidant effect of an organoselenium compound. Pharmacol. Res..

[57] Miao Y.F., Li J., Zhang Y.M., Zhu L., Chen H., Yuan L., Hu J., Yi X.L., Wu Q.T., Wan M.H. (2018). Sheng-jiang powder ameliorates obesity-induced pancreatic inflammatory injury via stimulating activation of the AMPK signalling pathway in rats: Basic Study. World J. Gastroenterol..

[58] Liang W., Menke A.L., Driessen A., Koek G.H., Lindeman J.H., Stoop R., Havekes L.M., Kleemann R., van den Hoek A.M. (2014). Establishment of a general NAFLD scoring system for rodent models and comparison to human liver pathology. PLoS ONE.

[59] Kleiner D.E., Brunt E.M., Van Natta M., Behling C., Contos M.J., Cummings O.W., Ferrell L.D., Liu Y.-C., Torbenson M.S., Unalp-Arida A. (2005). Design and validation of a histological scoring system for nonalcoholic fatty liver disease. Hepatology.

[60] Singh T., Allende D.S., McCullough A.J. (2019). Assessing liver fibrosis without biopsy in patients with HCV or NAFLD. Cleve Clin. J. Med..

[61] Pai S.A., Munshi R.P., Panchal F.H., Gaur I.S., Mestry S.N., Gursahani M.S., Juvekar A.R. (2019). Plumbagin reduces obesity and nonalcoholic fatty liver disease induced by fructose in rats through regulation of lipid metabolism, inflammation and oxidative stress. Biomed. Pharmacother..

[62] Wei J.L., Leung J.C.-F., Loong T.C.-W., Wong G.L.-H., Yeung D.K.-W., Chan R.S.-M., Chan H.L., Chim A.M., Woo J., Chu W.C. (2015). Prevalence and severity of nonalcoholic fatty liver disease in non-obese patients: A population study using proton-magnetic resonance spectroscopy. Am. J. Gastroenterol..

[63] Ghibaudi L., Cook J., Farley C., Van Heek M., Hwa J.J. (2002). Fat Intake Affects Adiposity, Comorbidity Factors, and Energy Metabolism of Sprague-Dawley Rats. Obes. Res..

[64] Suleiman J.B., Mohamed M., Bakar A.B.A., Nna V.U., Zakaria Z., Othman Z.A., Aroyehun A.B. (2021). Chemical Profile, Antioxidant Properties and Antimicrobial Activities of Malaysian *Heterotrigona itama* Bee Bread. Molecules.

[65] Bumrungpert A., Pingeesakikul T., Tirawanchai N., Tuntipopipat S., Lilitchan S., Komindr S. (2012). Effects of Ferulic Acid Supplementation on Lipid Profiles, Oxidative Stress and Inflammatory Status in Hypercholesterolemic Subjects. FASEB J..

[66] Prince S.M.P., Senthil Kumaran K. (2012). Preventive effects of caffeic acid on lipids, lipoproteins and glycoproteins in isoproterenol induced myocardial infarcted rats. Food Res. Int..

[67] Antuna-Puente B., Feve B., Fellahi S., Bastard J.P. (2008). Adipokines: The missing link between insulin resistance and obesity. Diabetes Metab..

[68] Febriza A., Ridwan R., As’Ad S., Kasim V.N., Idrus H.H. (2019). Adiponectin and Its Role in Inflammatory Process of Obesity. Mol. Cell. Biomed. Sci..

[69] Ryu J., Hadley J.T., Li Z., Dong F., Xu H., Xin X., Zhang Y., Chen C., Li S., Guo X. (2021). Adiponectin Alleviates Diet-Induced Inflammation in the Liver by Suppressing MCP-1 Expression and Macrophage Infiltration. Diabetes.

[70] Al Humayed S. (2016). Protective and therapeutic effects of *Crataegus aronia* in non-alcoholic fatty liver disease. Arc. Physiol. Biochem..

[71] Faheem S.A., Saeed N.M., El-Naga R.N., Ayoub I.M., Azab S.S. (2020). Hepatoprotective effect of cranberry nutraceutical extract in non-alcoholic fatty liver model in rats: Impact on insulin resistance and Nrf-2 expression. Front. Pharmacol..

[72] Bakour M., El Menyiy N., El Ghouizi A., Lyoussi B. (2021). Hypoglycemic, hypolipidemic and hepato-protective effect of bee bread in streptozotocin-induced diabetic rats. Avicenna J. Phytomed..

[73] Friedman S.L., Roll F.J., Boyles J., Bissell D.M. (1985). Hepatic lipocytes: The principal collagen-producing cells of normal rat liver. PNAS.

[74] Kyle T.K., Dhurandhar E.J., Allison D.B. (2016). Regarding Obesity as a Disease: Evolving Policies and Their Implications. Endocrinol. Metab. Clin. N. Am..

[75] Stec D.E., Gordon D.M., Hipp J.A., Hong S., Mitchell Z.L., Franco N.R., Robison J.W., Anderson C.D., Stec D.F., Hinds T.D. (2019). Loss of hepatic PPARα promotes inflammation and serum hyperlipidemia in diet-induced obesity. Am. J. Physiol. Regul. Integr. Comp. Physiol..

[76] Othman Z.A., Zakaria Z., Suleiman J.B., Nna V.U., Romli A.C., Ghazali W.S.W., Mohamed M. (2021). Bee Bread Ameliorates Vascular Inflammation and Impaired Vasorelaxation in Obesity-Induced Vascular Damage Rat Model: The Role of eNOS/NO/cGMP-Signaling Pathway. Int. J. Mol. Sci..

[77] Masella R., Giovannini C., Vari R., Di Benedetto R., Coni E., Volpe R., Fraone N., Bucci A. (2001). Effects of dietary virgin olive oil phenols on low density lipoprotein oxidation in hyperlipidemic patients. Lipids.

[78] Atrahimovich D., Vaya J., Khateb S. (2013). The effects and mechanism of flavonoid–rePON1 interactions. Structure–activity relationship study. Bioorganic Med. Chem..

[79] Jeepipallia S.P.K., Du B., Sabitaliyevich U.Y., Xu B. (2020). New insights into potential nutritional effects of dietary saponins in protecting against the development of obesity. Food Chem..

[80] Day C.P., James O.F. (1998). Steatohepatitis: A tale of two “hits”?. Gastroenterology.

[81] Zakaria Z., Othman Z.A., Nna V.U., Mohamed M. (2021). The promising roles of medicinal plants and bioactive compounds on hepatic lipid metabolism in the treatment of non-alcoholic fatty liver disease in animal models: Molecular targets. Arch. Physiol. Biochem..

[82] Watt M.J., Miotto P.M., De Nardo W., Montgomery M.K. (2019). The Liver as an Endocrine Organ—Linking NAFLD and Insulin Resistance. Endocr. Rev..

[83] Berlanga A., Guiu-Jurado E., Porras J.A., Auguet T. (2014). Molecular pathways in non-alcoholic fatty liver disease. Clin. Exp. Gastroenterol..

[84] Casas-Grajales S., Muriel P. (2015). Antioxidants in liver health. World J. Gastrointest. Pharmacol. Ther..

[85] Levonen A.L., Hill B.G., Kansanen E., Zhang J., Darley-Usmar V.M. (2014). Redox regulation of antioxidants, autophagy, and the response to stress: Implications for electrophile therapeutics. Free Radic. Biol. Med..

[86] Echeverría F., Valenzuela R., Bustamante A., Álvarez D., Ortiz M., Soto-Alarcon S.A., Muñoz P., Corbari A., Videla L.A. (2018). Attenuation of High-Fat Diet-Induced Rat Liver Oxidative Stress and Steatosis by Combined Hydroxytyrosol- (HT-) Eicosapentaenoic Acid Supplementation Mainly Relies on HT. Oxid. Med. Cell Longev..

[87] Peng C.H., Lin H.T., Chung D.J., Huang C.N., Wang C.J. (2018). Mulberry Leaf Extracts prevent obesity-induced NAFLD with regulating adipocytokines, inflammation and oxidative stress. J. Food Drug Anal..

[88] Zhao X.J., Chen L., Zhao Y., Pan Y., Yang Y.Z., Sun Y., Jiao R.Q., Kong L.D. (2019). *Polygonum cuspidatum extract* attenuates fructose-induced liver lipid accumulation through inhibiting Keap1 and activating Nrf2 antioxidant pathway. Phytomedicine.

[89] Wu K.C., Liu J., Klaassen C.D. (2012). Role of Nrf2 in preventing ethanol-induced oxidative stress and lipid accumulation. Toxicol. Appl. Pharmacol..

[90] Chen X.L., Gong L.Z., Xu J.X. (2013). Antioxidative activity and protective effect of probiotics against high-fat diet-induced sperm damage in rats. Animal.

[91] Chen Z., Tian R., She Z., Cai J., Li H. (2020). Role of oxidative stress in the pathogenesis of nonalcoholic fatty liver disease. Free Radic. Biol. Med..

[92] Cai D., Yuan M., Frantz D.F., Melendez P.A., Hansen L., Lee J., Shoelson S.E. (2005). Local and systemic insulin resistance resulting from hepatic activation of IKK-β and NF-κB. Nat. Med..

[93] Li Z., Yang S., Lin H., Huang J., Watkins P.A., Moser A.B., Desimone C., Song X.Y., Diehl A.M. (2003). Probiotics and antibodies to TNF inhibit inflammatory activity and improve nonalcoholic fatty liver disease. Hepatology.

[94] Das S.K., Balakrishnan V. (2011). Role of Cytokines in the Pathogenesis of Non-Alcoholic Fatty Liver Disease. Indian J. Clin. Biochem..

[95] Daly C., Rollins B.J. (2003). Monocyte chemoattractant protein-1 (CCL2) in inflammatory disease and adaptive immunity: Therapeutic opportunities and controversies. Microcirculation.

[96] Jensen V.S., Hvid H., Damgaard J., Nygaard H., Ingvorsen C., Wulff E.M., Lykkesfeldt J., Fledelius C. (2018). Dietary fat stimulates development of NAFLD more potently than dietary fructose in Sprague–Dawley rats. Diabetol. Metab. Syndr..

[97] Shen H.-H., Huang S.-Y., Kung C.-W., Chen S.-Y., Chen Y.-F., Cheng P.-Y., Lam K.-K., Lee Y.-M. (2019). Genistein ameliorated obesity accompanied with adipose tissue browning and attenuation of hepatic lipogenesis in ovariectomized rats with high-fat diet. J. Nutr. Biochem..

[98] Muhlbauer M., Bosserhoff A.K., Hartmann A., Thasler W.E., Weiss T.S., Herfarth H., Lock G., Scholmerich J., Hellerbrand C. (2003). A novel MCP-1 gene polymorphism is associated with hepatic MCP-1 expression and severity of HCV-related liver disease. Gastroenterology.

